# Challenges in the End‐of‐Life Care for Patients With Severe Persistent Mental Illness: A Case Series

**DOI:** 10.1111/psyg.70164

**Published:** 2026-04-07

**Authors:** Kaushadh Jayakody, Isha Bajaj, Doug Blomeley

**Affiliations:** ^1^ Institute for Mental and Physical Health and Clinical Translation (IMPACT) School of Medicine, Deakin University Geelong Victoria Australia; ^2^ Bendigo Health Bendigo Victoria Australia; ^3^ Faculty of Medicine Nursing and Health Sciences, School of Rural Health Monash University Bendigo Victoria Australia

**Keywords:** caregiver, end of life care, mental illness, older adults, palliative care

## Abstract

**Introduction:**

Despite reduced life expectancy and high rates of comorbidity, individuals with severe persistent mental illness (SPMI) face significant disparities in healthcare access and quality, which extends to palliative and end‐of‐life (EOL) care. Literature shows that this population is 3.5 times less likely to be referred to specialist palliative care services. Limited access to palliative and EOL care with suboptimal quality of care increases vulnerability and exacerbates suffering for both patients and their families.

**Methods:**

This retrospective case series examines the challenges encountered by older adults with SPMI, upon transitioning from curative to palliative approaches of care.

**Results:**

Analysis of three cases using deductive thematic analysis revealed key themes: delayed recognition of psychiatric treatment futility, late or denied access to palliative care, fragmented models of service delivery and ethico‐legal complexities. Referrals to palliative care occurred late—often days before death—thereby limiting opportunities for proactive care planning, caregiver support and preparation for EOL care.

**Conclusions:**

Early recognition of treatment futility and poor prognosis could have prompted earlier palliative care involvement, enhancing patient comfort and family support. A dynamic interplay between multimorbidity, frailty and dementia in the context of SPMI is observed, with these co‐occurring conditions collectively influencing clinical trajectories and service responses. This study emphasises the importance of improved clinician training, clearer referral pathways and integrated care models in addressing this disparity. Implementing these measures will aid in addressing longstanding inequalities and ensure individuals with SPMI receive appropriate and timely palliative and EOL care.

## 1 Introduction

The global population is ageing, and this trend is likely to continue [1, 2]. By 2050, the number of people aged 60 and over is projected to double, and those aged 80 or older to triple [3]. This shift brings a significant increase in years lived with illness. Older adults account for 23% of the global disease burden, largely due to chronic non‐communicable diseases [4, 5]. Mental illness is a major contributor to this burden, becoming increasingly prevalent and disabling with age [1, 4, 6, 7]. It increases vulnerability and increases the risk of physical comorbidities and dementia [6, 8, 9]. Multimorbidity, polypharmacy and reduced physiological reserve further impair clinical response, increasing frailty and mortality [1, 8, 10, 11].

Severe persistent mental illness (SPMI) encompasses enduring psychiatric conditions requiring long‐term treatment and causing significant disability [12, 13, 14, 15, 16, 17]. SPMI often follows a progressive course, with persistent or recurrent symptoms, diminishing treatment response and functional decline. This trajectory mirrors that of terminal physical illnesses, highlighting the need for a palliative approach focused on quality of life (QOL) and relief of suffering. Though by definition, these conditions include any psychiatric illness, schizophrenia, bipolar disorder, treatment‐resistant depression and anxiety disorders are most common [15, 17, 18, 19]. Dementia's classification is debated, but its neuropsychiatric features and psychiatric management align it with the SPMI construct [20].

Palliative care is a person‐ and family‐centred approach that aims to enhance QOL amid advanced illness and poor curative prospects [21, 22, 23]. Radbruch et al. (2020) add that palliative care ‘neither hastens nor postpones death’ and ‘affirms life’ [23]. End‐of‐life (EOL) care, a subset of palliative care, is typically delivered in the final months or weeks of life to individuals facing imminent death or those with advanced, incurable conditions or old age [24, 25]. Palliative care may begin early in the disease course, alongside curative treatments; whereas EOL care starts when curative interventions are no longer effective or desired and the care goal has shifted fully to comfort [2, 12, 26, 27].

Despite optimal treatment, some individuals with SPMI experience irremediable symptoms and progressive decline. Each unsuccessful treatment reduces the likelihood of future response, signalling a potential terminal trajectory [16, 20]. Chronic psychological distress can accelerate ageing, cognitive decline and mortality, further supporting the view that sustained psychiatric suffering imposes a physiological burden akin to terminal somatic illness [28]. Those with SMPI also present with other co‐morbidities such as long term physical illnesses which contribute to poorer health outcomes, difficulties in self‐management and reduced life expectancy [29].

At present, there are no established staging criteria or terminal‐phase protocols for those with SPMI. The Australia‐modified Karnofsky Performance Status Scale assesses functional status and terminal decline and has been validated across a range of diagnoses; however, its applicability in SPMI has not yet been examined [27, 30]. Furthermore, key frameworks, such as the WHO Mental Health Action Plan (2013–2030) and Australia's Exploratory Analysis (2019), exclude this population [22, 31]. The National Palliative Care Strategy (2018) highlights underserved populations but similarly omits those with SPMI [25].

A palliative approach to those with SPMI can significantly improve their care, autonomy and person‐centeredness [14]. In hospice and palliative care, decision‐making is often shared, reflecting autonomy as a mutual and relational process [12]. Similarly, world mental health policies emphasise early family engagement as crucial to ethical care honouring patient wishes [32]. Palliative psychiatry broadly aims to relieve suffering and enhance quality of life for patients and families beyond symptom control and more narrowly, refers to care for those dying from their mental illness [20].

Individuals with SPMI are known to face disparities in healthcare access and provision [19] Multimorbidity and long‐term conditions are highly prevalent within this population, exacerbating functional decline and intensifying care needs [1, 8, 29]. Most palliative care literature focuses on the role of psychiatry in supporting mainstream palliative patients [20, 33]. The voices of individuals with SPMI and their families regarding perceived EOL care needs are only beginning to emerge in the literature [11, 19, 29, 34]. For example, a thematic synthesis of previously published case studies exploring EOL care for those with SPMI identified five main themes [35]. Our case series tried to address this gap by presenting lived experiences of older adults with SPMI in regional Australia. For our three cases, a palliative approach was considered given the illness's severity and treatment futility. Our study is intended to enhance the existing knowledge base in this subject area and to advance understanding of how local clinical teams navigate the transition from curative to palliative approaches during EOL care when psychiatric illness is enduring and treatment‐refractory.

## 2 Aims and Objectives

### 2.1 Primary Aim

Our aim is to better understand how clinicians and systems respond when psychiatric treatment is no longer effective in relieving suffering or supporting recovery in people with SPMI, including how they navigate the transition towards palliative or comfort‐focused models of care.

### 2.2 Secondary Objectives

1. To examine how futility is identified and applied in psychiatric care for individuals with SPMI, particularly, in the context of treatment‐resistant illness and progressive functional decline
2. To explore how core palliative principles of relief of suffering, recognition of futility and holistic care are applied in psychiatric settings.
3. To identify clinical, systemic and ethico‐legal barriers to equitable palliative care access.
4. To understand how treatment goals shift from curative to palliative and how patient and family preferences inform this process

## 3 Methodology

### 3.1 Study Design

Our study employed a qualitative retrospective case series design, using deductive thematic analysis to explore its objectives. We used the Joanna Briggs Institute critical appraisal checklist for our case series to systematically evaluate our study quality, minimise bias, improve transparency and evidence synthesis [36]. This checklist informed the selection of cases with clearly documented diagnoses, explicit inclusion criteria, complete care trajectories from admission to death and detailed records of goals‐of‐care discussions and decision‐making processes.

### 3.2 Participants

The participants, all aged over 65, were patients of the Older Adult Unit within a psychiatry service at a large regional hospital in Australia between 2022 and 2024 (Table 1). This inpatient Older Adults Unit admits patients over 65 with psychiatric illnesses, such as depression, mania, anxiety disorders, psychosis, delirium and major neurocognitive disorder (dementia). Length of stay ranges from a few days to several months, depending on the psychosocial and medical complexity of each case. An average of one to three patients within this facility may progress to the palliative and EOL care pathway each year.

**TABLE 1:** Patient demographic, diagnosis and treatment history (from clinical records).

	Amy^a^	Beth^a^	Sara^a^
Age	92 years old	84 years old	91 years old
Sex	Female	Female	Female
Marital status	Widowed	Married	Widowed
Residence	Residential aged care facility	Home	Residential aged care facility
MTDM appointed (MTPD Act 2016) [37]	Yes	Yes	Yes
MTDM's relation to patient	Children (son and daughter)	Son	Daughter
Mental Health Diagnoses	Major depressive disorder with anxious distress, generalised anxiety disorder Major neurocognitive disorder (dementia)—presumed Alzheimer's and vascular aetiology	Major depressive disorder, generalisedgeneralised anxiety disorder Post‐traumatic stress disorder Major neurocognitive disorder (dementia)—frontal‐onset Alzheimer's disease	Major depressive disorder with psychotic features No formal dementia diagnosis
Mental Health and Wellbeing Act 2022 [38] status	Voluntary	Voluntary	Voluntary (community treatment order was revoked post discharge)
Duration of current admission	4 weeks	5 months	Residing in the community following a four‐week inpatient admission
Admission psychotropics	– Sertraline 50 mg (up‐titrated to 100 mg) – Risperidone 1 mg (up‐titrated to 1.5 mg)	– Sertraline 25 mg (up‐titrated to 200 mg) – Mirtazapine 30 mg – Quetiapine SR 100 mg (up‐titrated to 200 mg)	– Venlafaxine 150 mg – Mirtazapine 45 mg – Olanzapine 12.5 mg
Other pharmacological trials	– Tricyclic antidepressant‐ (poorly tolerated) – Olanzapine (minimal efficacy) – PRN haloperidol (minimal efficacy) – Regular benzodiazepines	– PRN (as required) quetiapine – PRN haloperidol – Aripiprazole – Regular benzodiazepines	
Other considerations	– Lithium (precluded by severity of renal impairment) – ECT		Regular benzodiazepines
Known duration of mental illness	Episodic‐requiring intermittent treatment across the lifespan.	6 years	Over 15 years
Past psychiatric admissions (Number and length of admission)	1 of 3 weeks duration	1 of 4 weeks duration	Over 10 of weeks–months in duration
Past treatments	– Antidepressants – Benzodiazepines (PRN)	– Antidepressants (combination) – Benzodiazepines – Antipsychotics (as augmentation)	– Antidepressants (combination) – Antipsychotics (as augmentation) – ECT
Currently case managed	Yes	Yes	Yes
Comorbidities	– Aortic valve disease – Osteoporosis – Osteoarthritis – Chronic kidney disease (Stage 3) – Hypertension	– Hypertension – Breast cancer (localised and resected)	– Hyperlipidemia – Hypertension – Chronic kidney disease (Stage 2) – Hypothyroidism
Age at the time of dementia diagnosis	89 years old	83 years old	N/A (not applicable)
Documented mental illness predating dementia	Yes (by decades)	Yes (by years)	Had an established history of severe mental illness requiring hospitalizations and treatments including ECT and vascular risk factors
Dementia severity: functional assessment staging tool (FAST) [39]	Moderate	Moderate	N/A
Frailty: clinical frailty scale (CFS) [40, 41]	Severely frail	Mildly frail	Moderately frail
AKPS score (as documented by palliative care team)	—	40	—
Advanced Care Directives (ACD) on EOL care wishes	No	No	No
Referred to palliative care	Yes	Yes	Not referred
Outcome of referral to palliative care	Not accepted	Accepted	N/A
Place of death	Residential aged care facility	Hospice	Residential aged care facility

^a^ Data were de‐identified. MTDM, Medical Treatment Decision Maker; MTPD, Medical Treatment Planning and Decisions; AKPS, Australia‐modified Karnofsky Performance Status; ECT, Electroconvulsive therapy.

### 3.3 Inclusion Criteria

- Aged 65 years or older.
- An established diagnosis of SPMI, defined in this study as a chronic, functionally impairing psychiatric disorder with limited responsiveness to standard treatment, including psychotic and major mood disorders. Although not traditionally classified under SPMI in this context, dementia and generalised anxiety disorder were included within the palliative psychiatry framework due to their high prevalence, chronicity, functional impairment and treatment resistance [7, 17, 20, 42].
- The underlying mental illness is deemed progressive, irreversible and treatment‐refractory and no longer amenable to curative treatments, as confirmed by a specialist psychogeriatrician.
- A documented shift in goals of care from curative (symptom reversal or restoration of function) to palliative (comfort‐focused care), in consultation with the hospital multidisciplinary team (which includes the psychiatry team, geriatricians and palliative care physicians) and family.
- Deemed psychiatrically terminal: having ongoing symptom burden unrelieved by adequate interventions, with deterioration in quality of life and function despite maximal therapeutic input. Maintenance medications continued for comfort and stability.
- Consent provided by a legally authorised representative (executor, medical treatment decision maker or next of kin)
- Not be eligible for a voluntary assisted dying pathway

### 3.4 Exclusion Criteria

- Under the age of 65 years
- Receiving psychiatric treatment with curative or rehabilitative intent, aimed at symptom reversal or functional restoration and recovery
- At risk of sudden or rapid death due to a terminal medical condition (e.g., cancer, end‐organ failure)
- This study's authors were not involved in treatment.

### 3.5 Data Collection and Analysis

For case summaries, two participants were selected from the inpatient setting and one participant from the outpatient setting. The participants recruited in this study were all deceased. Consent was obtained from the legal executors or from medical treatment decision makers, as per institutional ethics committee guidelines. Retrospective data was gathered from the clinical records (local electronic data recording system) using a standard data extraction sheet in an Excel spreadsheet. Data was de‐identified prior to storage by altering or removing direct (e.g., names, contact details, names of family members) and indirect identifiers (e.g., date of birth, occupation).

**FIGURE 1:**
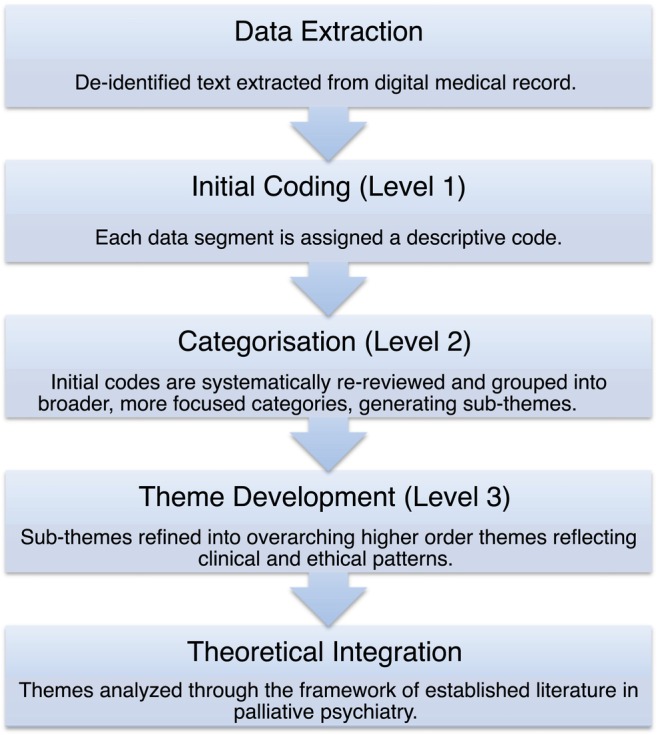
Deductive thematic analysis process.

Qualitative data techniques such as thematic analysis were used to gather information from inpatient clinical records over the span of a given patient's hospital stay (Figure 1). The thematic analysis provides a foundational core method for conducting many other forms of qualitative analysis [43]. The thematic analysis can be inductive or deductive. Deductive analysis is driven by the researchers' theoretical or analytic interest and may provide a more detailed analysis of some aspects of the data [44]. We used the thematic analysis deductive approach to develop codes and theoretical concepts [43, 44, 45, 46, 47]. Our theory‐driven approach was guided by pre‐existing theoretical frameworks, specifically established palliative care frameworks [48] and the biopsychosocial–spiritual model [49, 50]. Accordingly, data from inpatient clinical records were extracted relating to the integration of SPMI and EOL care, the identification of disparities in EOL care for individuals with SPMI and participants' trajectories and progress towards EOL care, including discussions with their families and the multidisciplinary team (including the psychiatry team, geriatricians and palliative care physicians), in order to identify common themes and key challenges in their care. All entries by the staff were then extracted. These included key issues observed by the staff, management issues recorded in the notes by the staff and incident records in the ward. The entries were from the psychiatry team (psychiatry nursing staff, nursing assistants, support workers, psychiatrists, psychiatry medical officers, occupational therapists, exercise therapists, physiotherapists, dieticians, psychiatry ward secretaries), general practitioners, geriatricians and palliative care physicians. Staff entries were then coded (Table 2).

**TABLE 2:** Themes and codes for theoretical concepts (clinical records).

Themes	Selected codes
Theme: challenges around acknowledging psychiatric treatment futility (attempts at active treatment continued despite significant clinical decline in the patients)	Amy^a^ ‘Do not think she is dying, and her trajectory remains unclear.’ (Palliative care team) ‘1on1 reassurance and music therapy provided with nil effect.’ (Mental health nursing); ‘Family was offered ‘a palliative approach or ECT for refractory distress.’ (Psychiatrist) ‘Family was worried about Amy's discharge destination and how anyone would cope with her distress and care needs.’ (Family) Beth^a^ ‘Remains very anxious. PRN given due to agitation and signs of distress—this had little effect. Staff have tried interventions such as music therapy and reading magazines however continued to be distressed.’ (Mental health nursing) ‘No further psychotropic treatment could be offered.’ (Psychiatrist) ‘She's not been the same since the accident.’ (Family) Sara^a^ ‘Residential aged care facility staff … requested Sara be admitted to hospital.’ (Residential aged care facility staff) ‘GP (General Practitioner) referred her to have blood tests and a CT‐brain.’ (GP via notes) ‘Emerging frailty and global decline … high risk for anesthetic complications due to severe high blood pressure before and after ECT treatment.’ (Psychiatrist)
Theme: challenges in incorporating patients' and families' preferences (the families expressed the following concerns while the psychiatric team continued to pursue active treatment interventions)	Amy^a^ ‘We just want Amy to be comfortable … not to suffer now.’; ‘Family declined further ECT.’ (Family) Beth^a^ ‘Stop all psychiatric medications … family would not want further procedures or investigations.’ (Family) Sara^a^ ‘Awareness of my mother's decline… let nature take its course as her quality of life now is poor.’ (Medical treatment decision maker)
Theme: system issues—barriers to equitable palliative care for people with SPMI–sub‐theme: Unconscious bias leading to possible stigma and discrimination (within this patient group, the presence of a SPMI rather than a terminal physical illness raises the question of whether this contributed to their not receiving palliative care services)	Amy^a^ ‘Local hospital refused transfer of care due to psychiatric origin of distress.’ (Psychiatrist) ‘Distress due to mental illness … this is not a terminal condition.’ (Palliative care team) ‘Psychiatry to guide benzodiazepine titration.’ (Palliative care team) Beth^a^ ‘Beth became more vulnerable from other patients.’ (Mental health nursing) Sara^a^ ‘Reluctant to utilise benzodiazepines due to Sara not presenting as anxious.’ (Residential aged care facility staff)
Theme: system issues—sub‐theme: fragmented communication (despite the psychiatry treatment team's request for palliative care involvement, the following considerations were documented as reasons for deviating from the initial plan)	Amy^a^ ‘No clear role for palliative care … recommend to reduce benzodiazepines.’ (Palliative care team) ‘Amy was denied hospice eligibility.’ (Psychiatrist) Beth^a^ (The statement below was documented in the clinical notes despite prior verbal clarification provided to staff) ‘Discontinuation of PRN antipsychotics as it may increase risk of vascular events’; ‘acknowledge staff anxiety.’ (Psychiatrist) Sara^a^ (A breakdown in communication was found, with the geriatrician formulating one plan while the psychiatry team proceeded with another) ‘Referred for blood tests and CT‐brain.’ (Geriatrician) ‘Family weren't made aware of the increasing risks previously’; will need authorisation from the Mental Health Tribunal.’ (Family)
System issues—sub‐theme: absence of clear referral criteria for palliative care (these comments suggest that there were no clear referral criteria for palliative care)	Amy^a^ ‘No clear role for palliative care.’ (Palliative care team) ‘Psychiatry to guide benzodiazepine titration.’ (Palliative care team) Beth^a^ ‘Beth became more vulnerable from other patients.’ (Mental health nursing) ‘Beth was referred to palliative care when her condition deteriorated further, and oral intake decreased.’ (Psychiatrist)
System issues—sub‐theme: limited staff training and support (despite the presence of treatment futility, staff had limited training to recognise and respond to it)	Amy^a^ ‘Reduce benzodiazepines due to risk of sedation.’ (Palliative care team) ‘This patient is not terminal.’ (Psychiatry registrar) Beth^a^ ‘Discontinuation of PRNs prescribed for the patient has increased staff anxiety.’ (Psychiatrist) Sara^a^ ‘Staff were reluctant to utilise benzodiazepines … and were feeling unable to manage Sara's presentation.’ (Residential aged care facility staff) ‘Visiting geriatrician discontinued oxazepam due to concerns about sedation.’ (Residential aged care facility staff)
Theme: ethical and legal challenges (sub‐theme: Ethical challenges; the treating teams faced ethical challenges as they weighted the potential benefits of continued intervention against the associated risks)	Amy^a^ ‘Amy was sedated soon after medications but woke up highly anxious and distressed.’ (Mental health nursing) ‘Family reduced visits as they felt it had increased her distress.’ (Family) ‘Re‐refer when patient deteriorates.’ (Palliative care team) ‘Reduce benzodiazepines due to risk of sedation.’ (Palliative care team) Beth^a^ ‘Staff asked family to reduce visits as her anxiety got worse and remained unsettled for a long time after they left.’ (Mental health nursing)
Theme: ethical and legal challenges (sub‐theme: legal challenges ‐ the treating team faced challenges in interpreting and implementing the various legislative frameworks relevant to the patient's care)	Amy^a^ ‘ECT required authorisation by the Mental Health Tribunal.’ (Psychiatrist) Beth^a^ ‘No advanced care directive had been prepared.’ (Psychiatrist) Sara^a^ ‘ECT required Mental Health Tribunal approval … unduly burdensome.’ (Medical treatment decision maker) ‘Oxazepam was discontinued … blood tests and CT‐brain ordered, but this is against medical treatment decision maker's plan.’ (Residential aged care facility staff)

^a^ Data were de‐identified.

In our qualitative data, a code was used as a basic analytic unit that was given a name that described what was being said or documented. Data from inpatient clinical records was extracted for all three patients independently. For each patient, all staff entries were extracted and coded (Level 1 coding; [45]). Initial codes were then re‐examined to search for emergent subthemes (Level 2 coding). Level 2 codes were further studied to develop highly refined subheadings and themes (Level 3 coding or themes). All codes were then reviewed again with reference to the original transcriptions (staff entries in clinical notes). Codes (Levels 1, 2 and 3) were iteratively adjusted to more appropriate codes. Themes were then expressed in longer phrases or sentences.

A theory‐driven approach allowed detailed examination of the data [44]. Rather than measuring the prevalence of themes, we examined how established issues manifested in complex clinical contexts and how they intersected with local organisational structures and legal frameworks. This interpretive approach supported our exploration of experience and nuance in these palliative care experiences of these patients.

### 3.6 Ethics Approval

Ethics approval has been obtained from the local ethics committee (application reference No 115368).

**Case Summary: Amy** ^**a**^

**Table:** 

Amy^a^ experienced profound depression and anxiety that proved resistant to comprehensive treatments, resulting in severe psychological distress and refusal of oral intake. This necessitated sedation for symptomatic relief and led to a marked deterioration in both mental and physical health, ultimately resulting in her death.

^a^ Data were de‐identified.

Amy, a 92‐year‐old grandmother, had lived in a residential aged care facility for the past year, needing some assistance with personal activities of daily living. Staying close to family and reading religious scriptures were deeply meaningful to her. She had a long‐standing history of recurrent major depressive disorder and generalised anxiety disorder, managed with antidepressants and benzodiazepines. Despite intermittent relapses, managed by her general practitioner, she never required psychiatric hospitalisation.

In early 2023, Amy was hospitalised twice for major depressive disorder with anxious distress. Despite antidepressant optimisation, antipsychotic augmentation and non‐pharmacological interventions, her symptoms and function deteriorated. Distressed by her worsening condition, her family reduced visits, as these appeared to exacerbate her anxiety. Benzodiazepines led to increased sedation with minimal relief. Her refusal to take medication made administration increasingly challenging. Amy experienced a progressive physical and nutritional decline, even losing interest in her religious readings. Her significant symptom burden, functional deterioration and minimal treatment response led to discussions with her family, one of whom was the medical treatment decision maker. Electroconvulsive therapy (ECT) was offered but declined due to its uncertain sustained benefit and potential adverse effects. The family prioritised comfort and adopted a palliative approach focused on symptom relief, not recovery. Given minimal response to other interventions, oral benzodiazepines were utilised to manage distress, despite associated sedation.

Palliative care referrals were made but declined, since Amy's distress was psychiatric. Oral benzodiazepines were further titrated to comfort. This resulted in increased sedation, but it was clinically necessary to manage Amy's persistent distress. As Amy's swallowing and alertness reduced, a second referral to palliative care was made, requesting consideration of hospice transfer for care in a calmer environment, away from an acute psychiatric ward. This request was also declined, with advice to switch to haloperidol. This proved ineffective and Amy's previous regimen was continued with family support.

The family request for Amy's transfer closer to home initially met with reluctance due to the psychiatric framing of her illness. But after several conversations with her general practitioner and residential aged care facility staff, she was transferred and passed away peacefully 2 days later, surrounded by family. The family acknowledged her terminal trajectory saying: ‘We just want mum to be comfortable, not suffering now’.

**Case Summary: Beth** ^**a**^

**Table:** 

Beth^a^ experienced progressively severe, treatment‐refractory agitated depression and anxiety that were unresponsive to specialised interventions. These psychiatric symptoms, exacerbated by medical complications, led to profound nutritional and functional decline, resulting in irreversible deterioration and eventual hospice admission for EOL care. Although Beth experienced medical illnesses, her psychiatric deterioration was the primary driver of her functional collapse and transition to EOL care.

^a^ Data were de‐identified.

Beth was an 84‐year‐old married grandmother living at home with her husband and was independent with personal activities of daily living. She was well supported by her family, who visited regularly and assisted with shopping and appointments. Her family described her as someone who valued family and independence and enjoyed gardening and knitting. Beth was admitted to hospital after a relapse of her pre‐existing major depressive disorder following surgery for a newly discovered breast cancer.

Beth had developed major depressive disorder, generalised anxiety disorder and post‐traumatic stress disorder following a car accident 6 years before. The severity of her symptoms had warranted care with secondary mental health services at the time. More recently, she had been diagnosed with frontal‐onset Alzheimer's disease.

During her five‐month admission, Beth's condition progressively deteriorated despite optimisation of her medications and non‐pharmacological input. Her progress was complicated by hospital‐acquired pneumonia, recurrent urinary tract infections and two significant falls—the latter resulting in a neck‐of‐femur fracture requiring surgery. While these events contributed to her overall frailty, her worsening psychiatric state, marked by agitation and emotional distress, remained the most persistent and debilitating issue. Her husband affirmed: ‘She's not been the same since the accident’.

The medical complications led to periods of intermittent delirium and Beth's depressive and anxiety symptoms worsened alongside reduced oral intake and a marked functional decline with minimal response to interventions. Doses of antidepressants and antipsychotics were regularly reviewed, adjusted and de‐prescribed if deemed ineffective. Regular benzodiazepines were commenced for symptom control.

Progress, treatment options and goals of care were discussed at a family meeting with the multi‐disciplinary team and all agreed that, despite ongoing medical interventions, Beth ‘was experiencing a rapid decline and was not good’. The family believed she ‘has no quality of life’, felt she was not benefiting from psychiatric medications and that ‘her wish would be to have no further procedures’. They wished ‘to stop all psychiatric medications’.

The palliative team confirmed Beth's deterioration and endorsed the withdrawal of curative care, recommending regular analgesia and Pro re nata (PRN; as required) midazolam and haloperidol for alleviation of distress.

Although hospice transfer was agreed upon, records show it was delayed by 72 h over concerns her psychiatric symptoms could disrupt care. A continuous subcutaneous infusion of midazolam was commenced 3 days following the transfer for symptom management. Beth died peacefully 4 days later.

**Case Summary: Sara** ^**a**^

**Table:** 

Sara's^a^ death was precipitated by a relapse of severe, progressive, treatment‐resistant major depressive disorder, leading to sustained refusal of intake and eventual multi system decline. ECT, although beneficial earlier in her illness, was discontinued due to mounting medical risks and a lack of sustained psychiatric recovery. Sara's family, one of whom was the medical treatment decision maker felt comfortable in deciding to move from curative to palliative treatment once the risks and benefits were openly discussed with them by the numerous medical clinicians involved. The shift from curative to palliative care not only aligned with Sara's lived trajectory but also the values expressed by her family. Sara's case highlighted challenges in conveying the family's palliative wishes across teams, highlighting the need for improved communication strategies.

^a^ Data were de‐identified.

Sara, a 91‐year‐old woman residing in a residential aged care facility, required assistance with activities of daily living. Sara had experienced recurrent episodes of major depressive disorder with psychosis, marked by swift mood deterioration, accompanied by minimal intake and nihilistic delusions, for over 15 years. In earlier stages, treatments, including ECT, were effective, achieving sustained remission, with preserved inter‐episodic functioning.

Over the years, relapse frequency increased and remissions became shorter. Since 2019, Sara required near‐continuous ECT, with acute or maintenance courses followed by rapid deterioration when treatment was spaced beyond fortnightly. By 2022, she had received approximately 90 ECT treatments and was also becoming increasingly physically frail. The progression of her illness showed a pattern of diminishing efficacy from previously effective interventions and a gradual functional decline.

In 2022, Sara was hospitalised as a compulsory patient for 4 weeks with a relapse of psychotic depression following a reduction in her maintenance ECT schedule. Some improvement followed an acute ECT course and she was discharged to her residential aged care facility.

At a multidisciplinary meeting with the ECT, anaesthetic and community psychiatry teams, the risks and benefits of ongoing ECT treatment were discussed with Sara's family. The team explained that Sara had experienced a progressive cognitive decline related to her SPMI, cumulative ECT effects and possible emerging dementia. Her family voiced concerns that ECT no longer yielded sustained benefit and Sara was experiencing increasing post‐treatment confusion and a prolonged recovery time with each successive course. Her daughter spoke of being ‘aware of my mother's decline’ and expected ‘nature to take its course as her quality of life now is poor’. A collective decision was made to discontinue ECT and adopt a palliative approach. Future relapses would be managed symptomatically, focusing on comfort and dignity. This represented a clinical and ethical pivot from active intervention to comfort‐based, dignity‐preserving care.

Over the next 4 weeks, clinical records show that Sara's mental state declined, prompting regular oxazepam for symptom relief. However, residential aged care facility staff struggled to manage her symptoms. A visiting geriatrician discontinued oxazepam over sedation concerns and initiated further investigations, including hospital‐based tests. This distressed Sara and her family, as it did not align with the previously agreed goals of care. A follow‐up meeting, with Sara's General Practitioner (GP), geriatrician and family, reaffirmed their preference for a palliative approach.

Sara was started on regular benzodiazepine and haloperidol as recommended by the treating psychiatrist. She died peacefully in her residential aged care facility 4 days later.

## 4 Results

Non‐pharmacological interventions including diversional therapy, music therapy and exercise‐based programmes were provided for all three patients. However, their levels of distress and comorbid cognitive impairment limited their capacity to engage meaningfully with structured psychological interventions.

### 4.1 Theme. 1: Challenges Around Acknowledging Psychiatric Treatment Futility

All three patients experienced persistent psychiatric symptoms without improvement, yet interventions continued despite clinical decline and transitions to palliative care occurred only after prolonged deterioration or family advocacy. These patterns are consistent with conceptual frameworks of futility and ‘terminal mental illness,’ in which repeated treatment failure and progressive functional decline indicate a transition from curative intent to a palliative focus [16, 51, 52, 53, 54]. In our cases, futility was often recognised late and implicitly, rather than through explicit staging or prognostic tools, reflecting broader calls for clearer criteria and guidance in the context of SPMI [55, 56, 57].

ECT remained under active consideration although Amy exhibited progressive physical and nutritional decline, with minimal response to interventions and died within days. Sara's ECT continued, despite increasing frailty and diminishing benefit. Even after ECT was stopped, medical investigations persisted, despite an agreed palliative plan. In Beth's case, multiple medication and non‐pharmacological strategies were trialled over 5 months without improvement, eventually prompting her family to request cessation of psychiatric medications. According to pre‐existing theories [48, 49, 50], the concept of ‘medical futility’ has been recognised in this population, however, despite this evidence, treatment for those patients in our case series appeared to have continued.

None of the patients in our case series developed a terminal physical illness that would have qualified them for palliative care. Therefore, the absence of a terminal physical illness created substantial barrier to palliative care access for all our three cases. For example, Beth's earlier infections were effectively treated, with no evidence of sepsis contributing to death (i.e., Beth's situation did not constitute a terminal physical illness).

### 4.2 Theme 2: Challenges in Incorporation of Patients' and Families' Preferences

In all three cases, families and medical treatment decision makers played a central role in care planning due to the patients' incapacity. Families expressed some understandings of the patients' values, which informed shared decision‐making and guided the transition from curative to palliative care. Pre‐existing theories [48, 49, 50] have already identified that the involvement of patients' families and their support networks plays an essential role in those facing EOL care. In situations where families are not involved or available and the patient's preferences are unknown, significant challenges arise for treatment planning and the provision of appropriate care. In our case series, Amy's family said, ‘We just want mum to be comfortable, not suffering now’, reflecting acceptance of her terminal trajectory.

Beth's family requested to ‘stop all psychiatric medications’ and emphasised she ‘would not want further procedures or investigations’, after prolonged inpatient care and continued decline.

Sara's medical treatment decision maker stated, ‘awareness of my mother's decline’ and chose to ‘let nature take its course as her quality of life now is poor’. They declined further ECT following a discussion of risks and benefits, opting instead for symptom‐focused care. By this stage, in all our three cases, the treating teams continued to pursue assertive treatment.

Our themes identified moral and ethical dilemmas encountered by treating teams in providing EOL care for those with SPMI, practically balancing the views and wishes of patient's families within clinical decision‐making processes. Previous research shows that those with SPMI, as well as their family members, are able to articulate clear EOL preferences when provided with adequate information and support and surrogate decision‐makers frequently advocate for comfort‐focused, value‐concordant care [34, 58, 59, 60, 61].

### 4.3 Theme 3: System Issues—Barriers to Equitable Palliative Care for People With SPMI

Prior research had identified system issues (e.g., disjointed clinical services) as a barrier to equitable access to palliative care for people with SPMI, in addition to systemic factors including diagnostic overshadowing, stigma and under‐recognition of terminal trajectories in those with SPMI [35, 62]. The main system‐level issues identified in our case series included possible unconscious bias contributing to stigma and discrimination, poor communication among staff, patients and their families, absence of clear referral criteria pathway for palliative care and lack of training and support for staff.

#### 4.3.1 Sub‐Theme: Unconscious Bias Leading to Possible Stigma and Discrimination

Amy's palliative care referrals were declined twice, with her distress attributed to mental illness and ‘not a terminal condition’, placing her condition outside the perceived scope of palliative care. Sara's residential aged care facility staff struggled to manage her, repeatedly ‘requesting hospital admission’, ‘ceased oxazepam’ and pursuing ‘blood tests and CT (computed tomography)‐ brain’—misaligned with agreed care goals. Beth's hospice transfer was delayed due to perceived behavioural risks. Nursing staff described her as becoming ‘more vulnerable from other patients’, while receiving EOL care in a psychiatric ward described as ‘loud and chaotic’. These responses illustrate the phenomena of diagnostic overshadowing and possible stigma; whereby psychiatric diagnoses disproportionately shape clinical judgement and limit the recognition of palliative care needs [11, 19, 35, 63]. Such discriminatory processes may contribute to a system in which those with SPMI are systematically excluded from services that are routinely accessible to others experiencing comparable levels of suffering and decline [17, 64].

#### 4.3.2 Sub‐Theme: Fragmented Communication

For Amy, the palliative care team recommended reducing benzodiazepines and substituting haloperidol, without adequately considering her documented history of poor tolerance to haloperidol or the family's stated preference for maintaining her comfort. Palliative care was declined on the grounds that her primary diagnosis was a mental health condition rather than a qualifying medical illness. In Sara's case, despite a shared palliative plan involving her family, medical investigations and hospital transfer were pursued. Concerns within the treating team regarding potential relapse contributed to delays in discontinuing ECT, despite minimal evidence of clinical improvement. Clinical records indicate that Sara's medical treatment decision maker reported not having been informed of the escalating risks associated with ECT in her case.

Fragmented communication across multiple services reflected broader system‐level deficiencies identified in the MENLOC and related studies, in which inadequate communication and ambiguous role delineation were associated with delayed referrals and predominantly reactive, crisis‐oriented care [35, 62, 64].

#### 4.3.3 Sub‐Theme: Absence of Clear Referral Criteria for Palliative Care

Amy's palliative care referral was declined twice and deferred, with advice to ‘re‐refer when deteriorates’, though she died a few days later in her residential aged care facility. Records show Sara was not referred during terminal decline. Beth was referred late, despite months of treatment‐refractory illness and her hospice transfer was further delayed and she remained vulnerable while receiving EOL care within an acute, high‐acuity psychiatric ward. The absence of established criteria for palliative care referral in those with SPMI appears to have contributed to delays and inconsistencies in decision‐making, despite clinical trajectories marked by functional decline, increasing frailty and treatment refractoriness—factors that, in other cohorts, were associated with elevated 1‐year mortality [1, 8, 57, 64].

#### 4.3.4 Sub‐Theme: Limited Staff Training and Support

Amy's distress appeared to be viewed as treatable and outside the scope of palliative care, with advice that ‘psychiatry should guide benzodiazepine titration’, despite her dying within days of referral. Sara's residential aged care facility staff showed reluctance to use prescribed benzodiazepines and initiated investigations not aligned with her care goals.

In Beth's case, cessation of as‐needed (PRN) medications appeared to heighten staff apprehension and further constrained the options available for managing her distress, despite the limited demonstrated efficacy of these interventions. Collectively, these observations suggest considerable uncertainty and anxiety among staff regarding the management of complex psychiatric symptoms within an end‐of‐life context, aligning with findings from prior research [12, 62, 65, 66]. Implementing joint training initiatives and shared protocols between mental health and palliative care services may help mitigate moral distress and support more consistent, goal‐aligned care [14, 20, 62, 64].

### 4.4 Theme 4: Ethical and Legal Challenges

Due to psychiatric illness and functional decline, none of the patients had capacity to make medical decisions. Records indicate that medical treatment decision makers were relied upon for care planning. In Sara's case, concerns about relapse delayed ECT cessation despite diminishing benefit and increasing risk.

Though none were treated under compulsory provisions, all patients received care through supported decision‐making involving families and medical treatment decision makers. Ethical tensions primarily concerned the challenge of balancing beneficence (alleviating suffering) with non‐maleficence (avoiding harm from over‐sedation or premature withdrawal of active treatment). These considerations had to be weighed alongside respect for autonomy, as articulated by families and medical treatment decision makers and the imperative of justice in ensuring equitable access to palliative care. Such dilemmas align with broader ethical analyses of palliative psychiatry and EOL within this population [13, 33, 67, 68, 69, 70].

Clinical records show that the Mental Health and Wellbeing Act 2022 (Victoria, Australia) was not formally invoked in any case. Although clinical documentation lacked explicit rationale, care proceeded collaboratively with medical treatment decision makers. The interface between the Mental Health and Wellbeing Act 2022 and the Medical Treatment Planning and Decisions Act 2016 (Victoria, Australia) can generate ambiguity when psychiatric and medical decision‐making converge. This uncertainty may contribute to clinicians' reluctance to conceptualise SPMI as potentially terminal and to initiate palliative care, despite legislative commitments to supported decision‐making and least restrictive practice.

Sara's ECT required Mental Health Tribunal approval. Her medical treatment decision maker found the process ‘unduly burdensome’ given the treatment's declining benefit and increasing risk. Despite an agreed plan, Sara's oxazepam was discontinued and diagnostic tests ordered—contradicting medical treatment decision maker directives. In Beth's case, the team sought an Advanced Care Directive, but none existed and family input guided decisions.

## 5 Discussion

Our study identified key themes including limited recognition of psychiatric treatment futility, delayed or denied access to palliative care, fragmented service models (unconscious bias leading to possible stigma and discrimination, fragmented communication, absence of clear referral criteria for palliative care, limited staff training and support), challenges in incorporating patients' and families' preferences and ethico‐legal complexities. Palliative care referrals were made very late which left little time for planning ahead, supporting caregivers, or preparing for EOL care. In all three cases, patients showed persistent psychiatric symptoms that did not respond to treatment, with no indications of personal or clinical recovery, necessitating a transition from curative to palliative care. However, this shift came late in hindsight, all patients died within days of referral. Neither patients nor families had adequate time or support to prepare for EOL care, a core component of palliative care [12]. Early palliative input could have enabled proactive symptom management, realistic goal setting, psychosocial‐spiritual care and acceptance of dying, avoiding futile treatments and growing hopelessness [2, 55, 62]. Consistent with the findings of the MENLOC study [35, 62], our cases demonstrate fragmented care pathways, insufficient cross‐sector collaboration and persistent difficulties in recognising physical deterioration when psychiatric symptoms predominate during clinical encounters. Similar to our findings, previous literature [71, 72] exploring these areas identified several themes including diagnostic delay, challenges regarding decisional capacity of individuals, professional dilemmas in making treatment decisions, medical futility, absence of support networks for those with SPMI and issues in provision of care. Another study [34], exploring the EOL experiences among those with SPMI found that having healthy relationships was a highly valued priority during this end stage of care. In our study, incorporation of patients' and their families' wishes and preferences regarding the care of their loved ones was identified as another challenge faced by treating teams when making ethically and morally decisions about patient care. Results from a cohort study [64] show that those with SPMI experience reduced life expectancy compared with those without SPMI and those with SPMI have more unscheduled health care episodes, spending more time in hospital and receive lower rates of palliative care. Similarly, our study highlights the difficulties in accessing palliative care for those with SMPI receiving end of life care. A systematic review found that patients with SPMI received specialist palliative care at a rate of 0.5%, compared to 1.72% in the general population. The same study also analysed decedents who died from non‐sudden causes, revealing that 8.5% of patients with psychotic disorders received palliative care, compared to 14.9% of general patients [12].

All patients in our case series had comorbid dementia, but their functional status did not meet criteria for end‐stage or terminal dementia [2, 39]. Moreover, they did not demonstrate the characteristic symptoms of terminal physical illness—such as pain, dyspnea, or nausea—which may have further obscured recognition of an underlying terminal decline [27, 73]. Instead, their clinical trajectory was marked by persistent psychological suffering, treatment refractoriness and functional deterioration. These features appeared to be more suggestive of worsening of the underlying psychiatric illness, compounded by frailty and physical comorbidities [1, 7, 8, 9, 74, 75]. Sedation was initially utilised to manage refractory psychiatric symptoms, but as deterioration progressed, continuous sedation became necessary to ensure comfort and alleviate suffering, consistent with palliative care principles [76, 77, 78].

Although SPMI is rarely classified as terminal, when marked by irreversibility, progressive decline and treatment non‐response, it mirrors the trajectory of terminal somatic illnesses [16, 24, 25, 26]. Psychiatric disorders are also linked to increased cognitive decline and dementia risk, reinforcing their potentially terminal nature [9, 55, 79]. Population‐level evidence from prior research indicates that those with SPMI experience substantial utilisation of unscheduled healthcare services and highly variable access to specialist palliative care in the final year of life [64]. These patterns highlight persistent structural inequities and signal missed opportunities for earlier intervention.

Palliative psychiatry accepts that some cases of SPMI may be irremediable, warranting a shift from curative intent to relieving suffering and preserving personhood [14, 20]. Medical futility may be quantitative, when treatment fails to achieve its intended physiological effect, or qualitative, when recovery is no longer feasible despite intervention [51, 52, 53]. In psychiatry, futility is often misinterpreted as therapeutic abandonment, though it more accurately signals the need to withdraw aggressive, non‐beneficial treatments [20, 51, 54]. Repeated treatment failure and functional decline are key indicators of futility, yet recognition is limited by the absence of clinical staging models and prognostic tools [16, 53, 55, 56]. Persisting with futile interventions may cause more harm than benefit; thus, decisional capacity should not delay reconsideration of care goals [54, 67]. Psychiatry's limited engagement with death and dying further reinforces the misconception that palliative care opposes recovery, when in fact it fosters hope, dignity and quality of life beyond symptom control [13, 14, 20, 55].

To the best of our knowledge, there is no established consensus within psychiatry regarding the definition of terminal or advanced illness. Berk et al. (2012) argue that some psychiatric disorders progress through stages, with early stages more responsive to treatment and advanced stages showing persistent symptoms and reduced efficacy due to progressive neurobiological changes [55]. Similarly, Lopez et al. proposed criteria, including poor prognosis, non‐response, continuing decline and terminal trajectory; however, there is no operational guidance [51]. Nonetheless, functional decline, frequent hospitalizations, escalating care needs and refractory symptoms should prompt a palliative care referral [2, 80]. Prognostic tools, such as the ‘surprise question’(Would you be surprised if this patient died within the next year?), may support such assessments [80, 81]. Consistent with our case series, Burton et al. found elderly psychiatric inpatients with comorbid dementia, or two or more admissions, had elevated one‐year mortality risks [57].

Older patients with SPMI often face multimorbidity, frailty and polypharmacy, which together lower treatment tolerance and clinical response and increase risks [1, 8, 82]. While younger patients may better tolerate and benefit from aggressive interventions, older patients may need early palliative consideration [2, 83]. Findings from Carswell et al. demonstrate that those with SPMI face significant difficulties in self‐managing co‐occurring physical and mental health conditions, challenges that have important implications for clinical deterioration and patterns of help‐seeking [29].

Studies including our study highlight multiple barriers to EOL discussions and access, including stigma, staff inexperience, diagnostic overshadowing, behavioural challenges, assumptions about capacity and destabilisation, communication difficulties and lack of social supports [12, 13, 19, 63, 65, 66]. Yet, research shows that people with SPMI can participate in these decisions and support the appointment of a surrogate when capacity is lacking [58, 59, 60, 61]. Several studies have explored the reasons behind disparities in care for patients with SPMI. They highlight that non‐psychiatric medical staff often lack adequate training to communicate effectively with SPMI patients and may assume these patients have limited real or perceived capacity for medical decision‐making—creating significant barriers to providing high‐quality palliative and end‐of‐life care. Conversely, mental health teams report feeling uncomfortable and insufficiently knowledgeable about palliative care, which makes even basic palliative care decisions challenging [11, 13, 34, 71, 74], highlights the importance of open and explicit discussion of both mental illness and EOL concerns among those with SPMI, their relatives and healthcare professionals—this study further identifies relational continuity, effective communication and reduced stigma as central components of high‐quality care [34]. Likewise, population‐level data from Coffey et al. (2025) indicates that limited access to specialist palliative care is associated with increased utilisation of unscheduled care, mirroring the late referrals and crisis‐driven trajectories observed in our cases [64].

Ethico‐legal barriers may further hinder access to palliative care. Patients in this study appeared to have faced barriers to palliative care due to the psychiatric nature of their suffering, highlighting concerns of justice, equity and access. A central ethical dilemma was whether to pursue palliative care based on poor prognosis or continue curative efforts despite suffering. However, when interventions do not benefit but instead prolong suffering, shifting focus to palliative care is ethically warranted, upholding beneficence and non‐maleficence [67, 68]. In SPMI, palliative and EOL care also aim to enhance quality of life and must follow the same ethical principles as somatic care: autonomy, beneficence, non‐maleficence and justice [69, 70]. Capacity assessments in SPMI are complex; patients are often presumed incapable solely based on their diagnosis; thus refusals frequently lead to paternalistic or involuntary treatment [13, 33]. Death in institutional psychiatric care mandating coronial inquiry further contributes to fear of litigation and deters staff from initiating palliative care [13, 33, 84]. Advanced care directives could support planning but often reflect general psychiatric rather than EOL preferences, are underutilised and may lack enforceability [12, 14, 75].

In Australia, the Mental Health and Wellbeing Act (2022) and Medical Treatment Planning and Decisions Act (2016) govern some of the areas, such as psychiatric and medical decisions. While the Medical Treatment Planning and Decisions Act (2016) supports value‐aligned care, the Mental Health and Wellbeing Act (2022) permits compulsory treatment based on risk when patients decline treatment or are unable to make decisions, creating ambiguity. Balancing patient autonomy with the clinician's duty to provide care and prevent harm emerges as a common ethical challenge in this scenario. None of the patients in the study refused care; treatment plans were collaboratively formed with families and medical treatment decision makers. Coercive treatment may have extended life but was unlikely to restore meaningful quality of life and thus would breach ethical and legal standards. As is known, coercion has a negative impact on quality of life and clinical outcomes [85, 86]. Instead, palliative models, endorsed by substitute decision‐makers, better respect dignity and autonomy and the least restrictive approach, in line with the Mental Health and Wellbeing Act (2022).

As observed in our case series and consistent with the literature, families often align with patient values [13]. However, many individuals with SPMI may lack such support [12, 13]. In these cases, clinicians must advocate for patients, building on respect, honesty and compassion [13]. Morita et al. argue that only patients can decide when suffering becomes intolerable, aligning with the Lancet's concept of Serious Health‐Related Suffering, advocating for care based on need, not diagnosis [5, 77]. According to previous research, patients and carers expressed frustration with the poor care they experienced during the final weeks of life, feeling abandoned, unheard and ignored by clinical staff. They reported being often excluded from advanced care planning and decision‐making due to the stigma and prejudice surrounding their SPMI diagnosis. They urged healthcare professionals to adopt the right attitude and approach, emphasising the need to see them as individuals, not just their diagnosis [65].

Recognising terminal psychiatric trajectories appears to be critical, but caution is needed against using it to justify resource rationing [52]. The term ‘palliative treatment’ in psychiatry may carry negative connotations and should be used cautiously to avoid conveying a sense of hopelessness or therapeutic nihilism [14, 55].

The principle of parity of esteem supports the obligation to provide equitable standards of care for those with SPMI and those with physical health illness [16, 62]. Though palliative care goals align with psychiatry, their practical integration is limited [12, 15, 33, 62, 65]. Collaborative models with geriatric, palliative and primary care clinicians, supported by shared frameworks and cross‐training, are key to psychiatric palliative care [12, 14, 20, 65]. These can improve quality of life, reduce suffering and enhance family satisfaction with care and promote timely, planned support for people with SPMI at the end of life [2, 20, 62, 64]. Palliative care is a recognized component of the human right to health, reinforcing the universal right of individuals with SPMI to dignified, nonstigmatized care [13, 17]. Previous studies highlight the suboptimal care experienced by SPMI patients and offer suggestions to reduce this disparity. These included simple steps such as involving the expertise of consultative‐liaison psychiatrists to bridge the gap between medical and mental health teams, correcting existing misconceptions, emphasising the importance of advanced care planning, recognising social needs and addressing suffering and ensuring ongoing access to psychiatric treatment and long‐term caregiving [87, 88].

## 6 Limitations and Future Research Considerations

There are several limitations in our study. The small, ethnically homogeneous sample comprising three elderly, frail patients with comorbid dementia limits generalisability. The universal presence of dementia may have influenced outcomes, although its distinct contribution to prognosis in these cases remains unclear. Carers' perceptions of preparation for the EOL phase and bereavement support were not specifically explored, although access to such support appeared limited. The patients had strong family advocates who supported complex EOL decisions, which may not reflect the experience of all individuals.

Selection bias may have been introduced by the retrospective nature of the case series, which relied on clinical documentation that may have been incomplete or inconsistent. The authors' dual role as treating clinicians may also have introduced bias in data interpretation and thematic analysis. Additionally, eligible patients who were not under the authors' care may have been excluded, further compounding the potential for selection bias.

The Mental Health and Wellbeing Act (2022; Australia) was not formally applied in any of the cases, with the reasons for this undocumented, leaving the legal reasoning and application underexplored. Interviews with families or patient‐reported outcomes were lacking, thus limiting the study's ability to capture the perspectives of those most affected. Additionally, clinical documentation primarily captured medical and pharmacological interventions, while psychosocial, spiritual, cultural and other non‐medical aspects of care were inconsistently recorded and are therefore likely to be under‐represented. Additionally, the absence of input from palliative care or geriatrics may have skewed findings towards a predominantly psychiatric perspective, overlooking the broader interdisciplinary considerations in EOL care.

We applied the deductive approach—one limitation of the deductive approach is that it tends to produce a less rich description of the overall data. A future qualitative study with a larger and more diverse sample should explore the perspectives of patients, families and multidisciplinary teams—including mental health, geriatric, palliative and hospital management—using interviews, questionnaires and clinical record reviews [46]. Rigorous, high‐quality qualitative research is needed in this area and encouragingly, such work is beginning to emerge. Future studies should meaningfully involve those with SPMI in the design, conduct and dissemination of the research, ensuring that their priorities guide the process. This approach is required for producing findings that are relevant to their lived experiences and applicable to the development of service improvements. Further research should also examine the role of interdisciplinary training, advanced care directives, palliative sedation, structured palliative pathways and psycho‐palliative teams across diverse care settings.

## 7 Conclusions

In all three cases, delayed recognition of futility and impending terminal outcomes, combined with diagnostic bias and systemic shortcomings, constrained timely access to palliative care. As discussed above, this study has several limitations and its findings should therefore be interpreted with caution and not over‐generalised.

Individuals with SPMI are among the most vulnerable in mental healthcare, facing increased risk of both therapeutic neglect and overly aggressive interventions with limited benefit. Clinicians struggle to define futility in psychiatric contexts, largely due to the absence of validated tools, prognostic markers and training. Diagnostic overshadowing, systemic stigma, fragmented communication and lack of established referral criteria further compound access barriers.

Nevertheless, families and medical treatment decision makers played a decisive role in guiding care goals, consistently advocating for comfort and dignity. Their involvement highlights the feasibility and ethical necessity of collaborative, person‐centred care, even when capacity is impaired.

Our study advocates for the development of psychiatric palliative care frameworks, interdisciplinary training and greater legislative clarity on EOL scenarios for individuals with SPMI and inclusion of patients and carers in care delivery and planning. It further highlights the need to address multimorbidity through service models that recognise and respond to the complex interplay between chronic physical and mental health conditions in later life.

Ultimately, recognising terminal psychiatric trajectories is not therapeutic nihilism but an ethical imperative to alleviate suffering, uphold dignity and integrate mental health within the broader continuum of palliative care.

## Funding

The authors have nothing to report.

## Disclosure

The research project was approved and assessed by the Royal Australian and New Zealand College of Psychiatrists' Scholarly Projects Committee.

## Ethics Statement

Ethics approval has been obtained from the local ethics committee (application reference No 115368).

## Conflicts of Interest

The authors declare no conflicts of interest.

## Data Availability

The data that support the findings of this study are available on request from the corresponding author. The data are not publicly available due to privacy or ethical restrictions.
